# Transport-related walking among young adults: when and why?

**DOI:** 10.1186/s12889-020-8338-0

**Published:** 2020-02-18

**Authors:** Behrang Assemi, Renee Zahnow, Belen Zapata-Diomedi, Mark Hickman, Jonathan Corcoran

**Affiliations:** 1 School of Built Environment, Queensland University of Technology (QUT), Gardens Point, 2 George St, Brisbane, QLD 4000 Australia; 2 School of Social Science, University of Queensland, St Lucia, QLD 4072 Australia; 3 School of Global, Urban & Social Studies, RMIT University, 124 La Trobe Street, Melbourne, VIC 3000 Australia; 4 School of Civil Engineering, University of Queensland, St Lucia, QLD 4072 Australia; 5 School of Earth and Environmental Sciences, University of Queensland, St Lucia, QLD 4072 Australia

**Keywords:** Physical activity (PA), Walking, Active travel, Activity node, Smartphone, Global positioning system (GPS), Directed graph

## Abstract

**Background:**

The existing smartphones’ technology allows for the objective measurement of a person’s movements at a fine-grained level of geographic and temporal detail, and in doing so, it mitigates the issues associated with self-report biases and lack of spatial details. This study proposes and evaluates the advantages of using a smartphone app for collecting accurate, fine-grained, and objective data on people’s transport-related walking.

**Methods:**

A sample of 142 participants (mostly young adults) was recruited in a large Australian university, for whom the app recorded all their travel activities over two weekdays during August–September 2014. We identified eight main activity nodes which operate as transport-related walking generators. We explored the participants’ transport-related walking patterns around and between these activity nodes through the use of di-graphs to better understand patterns of incidental physical activity and opportunities for intervention to increase incidental walking.

**Results:**

We found that the educational node — in other samples may be represented by the workplace — is as important as the residential node for generating walking trips. We also found that the likelihood of transport-related walking trips is larger during the daytime, whereas at night time walking trips tend to be longer. We also showed that patterns of transport-related walking relate to the presence of ‘chaining’ trips in the afternoon period.

**Conclusions:**

The findings of this study show how the proposed data collection and analytic approach can inform urban design to enhance walkability at locations that are likely to generate walking trips. This study’s insights can help to shape public education and awareness campaigns that aim to encourage walking trips throughout the day by suggesting locations and times of the day when engaging in these forms of exercise is easiest and least intrusive.

## Background

*I travel not to go anywhere, but to go. I travel for travel’s sake. The great affair is to move.* [1]

Regular physical activity (PA) lowers the risk of developing chronic diseases (cardiovascular disease, type 2 diabetes and some types of cancer), cognitive decline and dementia, improves musculoskeletal health and contributes to weight management [2–5]. National public health authorities recommend at least 30–60 min of moderate-to-high intensity PA on most weekdays for adults for maintaining a healthy life style [6–8]. Still, 23% of the adult population worldwide, and 44% in Australia, are not sufficiently active to accrue health benefits [6, 9]. Australian studies show that low levels of PA are responsible for 10–20% of the burden of related diseases [10, 11]. Overall costs related to physical inactivity in Australia were estimated at AU$805 m in 2013 [12], with 16,178 deaths attributed annually to being physically inactive [11].

The literature suggests that even small increases in PA can improve people’s health status [13, 14]. Incidental physical activity — PA accumulated through normal daily activities un-associated with exercise goals, such as walking for transport purposes — is attracting the attention of researchers and policy makers as a means to improve the overall health status in communities [15, 16]. Research has shown that increasing PA for transport (i.e., active travel), positively contributes to people’s health and happiness [14, 15, 17, 18]. Active travel constitutes any kind of travel between places through walking, cycling or other non-motorised modes of transport [15].

Walking is the most common form of active travel in Australia, with 3.5% mode share for work or education trips [19]. While this is a small share, there is high potential to replace private motor vehicle trips with active travel as 25% of trips are under 5 km [19]. Walking uses approximately 3.5 times the energy used when sitting; this is equivalent to the energy consumed during moderate-intensity PA [11]. Replacing private motor vehicle trips with public transport can also accrue important health benefits from walking to access/egress transit and improved air quality [20, 21]. However, the literature notes declining rates of transport-related walking in the past 20 years [22, 23]. This decline may be, in part, related to urban form (for example, availability and frequency of transit, street connectivity and land-use mix), reliance on private vehicles and longer commutes between home and work [11, 24, 25]. This suggests that urban areas can be designed to maximise opportunities for transport-related walking to support improvements in public health and wellbeing [26–28].

Conventionally, studies on urban form and active travel have relied on self-report questionnaires or travel diaries to capture active travel patterns [24, 29]. The data collected with such instruments are subject to recall bias and bias from misclassification of activities reported by participants [30, 31]. Short trips, often walking trips under 10 min, are usually missing in the data collected by these instruments [32]. To overcome these issues, studies have used wearable sensors (e.g., pedometers and accelerometers), which allows for more accurate data collection on *time engaged in PA* compared to time engaged in sedentary activity [33].

To date, studies have mostly investigated walkability of *residential neighbourhoods* in relation to active travel [34]. Active travel may occur in multiple urban settings (e.g., work, education and home neighbourhoods); yet, the literature has mainly focused solely on active travel in the home neighbourhood environment [33]. Hence, we have limited empirical evidence to support an understanding of **when** and **why** active travel is most likely to occur; limiting capacity for effective, targeted urban design strategies. This is mainly due to a lack of tools to support fine-grained data collection to investigate the association between urban form and PA.

Recently, transport authorities have used smartphone applications based on global positioning system (GPS) technology for collecting data on people’s travel behaviour to complement the data collected by conventional methods (e.g., travel diaries) [35, 36]. This is especially important as the possibility of mapping active travel to urban form in diverse settings enables us to better understand people’s active travel patterns [37]. The GPS-enabled technology allows for the objective measurement of a person’s movements at a fine-grained level of geographic and temporal detail, and in doing so, it mitigates the issues associated with self-report biases and lack of spatial details [31, 38]. The widespread use of smartphones has been shown to dramatically simplify the data collection process [38, 39], allowing for large-scale studies of people’s PA and health behaviours [30].

Therefore, our study investigates potential advantages of smartphone-assisted data collection to study people’s active travel patterns focusing on transport-related walking during a person’s regular daily routine. An understanding of such patterns can inform urban design and indicate where PA-facilitating urban form features such as sidewalks can have the greatest potential to impact population health. We build upon recent work (e.g., [30, 31, 37, 38, 40]) by automatically collecting continuous data on young people’s active travel behaviour, and exploring the timing and location of transport-related walking for our sample. We identify eight main activity nodes where transport-related walking originates from or leads to, including education, home, work, shopping, health/wellbeing, eat/drink, changing mode and other. We study transport-related walking trips between these activity nodes at five different time-slots throughout the day, using di-graphs, to reveal potential time-specific patterns.

The remainder of the paper is structured as follows. In the next section, we present the methods of data collection (including the characteristics of our smartphone application and the survey design) as well as the analysis and modelling techniques. Next, we present the results of a study of 142 predominantly young adults in Brisbane, Australia. Finally, we discuss the implication of the study results for transport-related walking and provide a set of concluding remarks in the last section.

## Methods

### Data collection

In this study, we designed and implemented a smartphone application, namely ATLAS II^1^, to collect data on participants’ transport-related PA, specifically during their travel activities. The application automatically records all movements of its user, while silently working in a smartphone’s background (it does not require any interaction with the user when recording their movements). This approach avoids reporting bias that can arise either consciously or unconsciously. Therefore, it enables the capture of “normative,” incidental PA; such PA is sometimes so benign and routinised that individuals can forget to document in self-report studies using conventional questionnaires. The use of a smartphone application for data collection provides an efficient way to holistically capture the characteristics and dynamics of people’s mobility and transport-related walking over time and across places. The application also incorporates a customisable socio-demographic questionnaire to collect relevant data when each participant runs the application for the first time. The application is developed for both iOS and Android, and it is publicly available on App Store and Google Play Store for download.

When the phone is carried by its user beyond a customisable distance threshold (10 m in this study), the application automatically starts recording the user’s movements as a trip by logging the corresponding data locally on the user’s smartphone. The application stops recording the trip when the user remains stationary beyond a customisable time threshold (2.5 min in this study). The first threshold is set to mitigate potential privacy concerns of research participants [41], and the second threshold is set based on the dwell time between two subsequent trips which has commonly been considered by previous research [42, 43].

Incorporating a measure of dwell time means that the application breaks down the recorded movements and stores them as separate trips, when it detects some idle time — larger than the specified threshold — between movements. The application records accurate traces of all movements of the user using the combination of GPS, global system for mobile communications (GSM) and Wi-Fi signals for the whole duration of data collection. Each log in the recorded data set, which is captured every 2 s on average while the user is moving, includes accurate latitude, longitude, instant speed, logging accuracy, heading and time-stamp.

A trip, as recorded by this application may consists of multiple single-modal trip-legs, given there is not any significant idle time between them. To enable a more accurate identification of transport-related walking while post-processing the recorded data, the user is asked to reveal a few attributes about each recorded trip, mainly the mode(s) and purpose of the corresponding trip. This is handled by requesting the user to view and label each recorded trip in the application, at the end of each day. The data, then, are uploaded to a server and become accessible for analysis upon the user’s approval.

**Fig. 1:**
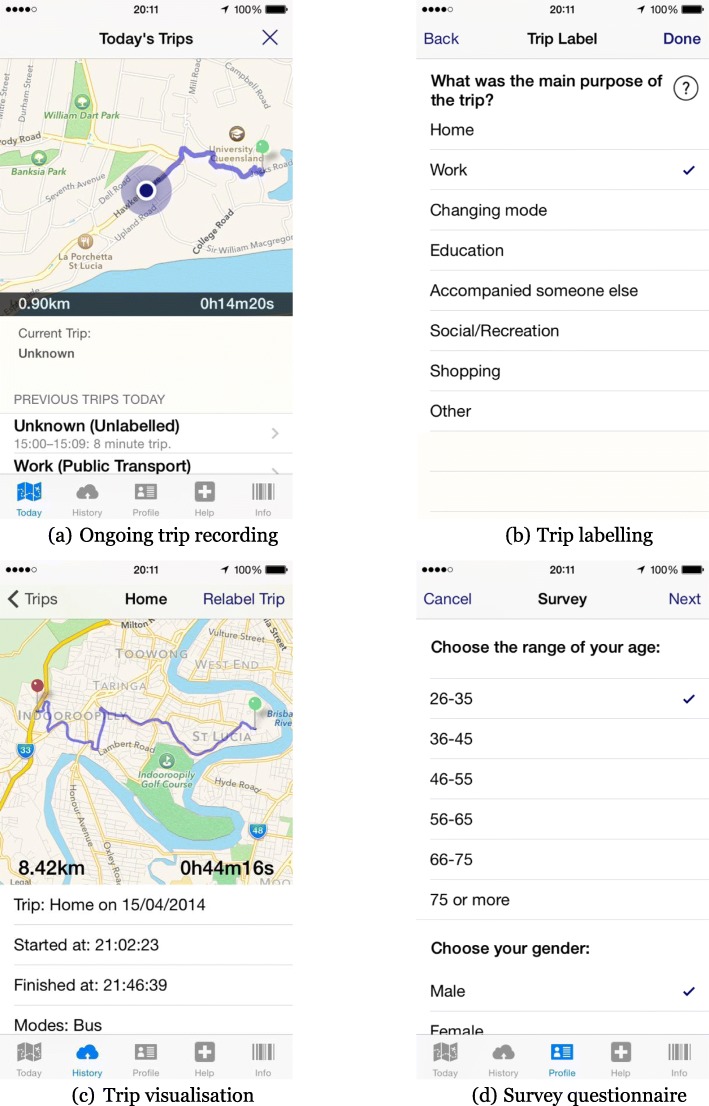
Smartphone application screenshots

Figure 1 illustrates selected screenshots of the smartphone application. Figure 1 (a) shows the main tab of the application while the application is recording a trip. On this tab, the application illustrates the ongoing recording process (including the trip’s complete trajectory on a map along with the total time and distance travelled in the trip), and the current location of the user. To assist the user with identifying their trip attributes, the application visualises the trajectory of each trip on a map with the origin address, destination address, start time, finish time, and total distance travelled. Figure 1 (b) shows how users specify their trip attributes, while Fig. 1 (c) illustrates the relevant recorded trip. Finally, Fig. 1 (d) shows the survey questionnaire implemented in the application to collect the socio-demographic details of the user along with any other relevant information.

As the smartphone application collects accurate data on the location of its users, it is important to incorporate explicit policies and technical settings to protect the users’ privacy. First, the proposed data collection approach using the smartphone application is governed by an ethics application reviewed and approved by the University of Queensland’s Human Research Ethics Committee. As explicitly mentioned in the application, the proposed approach could only be used for research purposes, while no identifiable details about participants are collected or kept throughout the process.

Second, the accuracy of location data captured by the application as well as the time and distance threshold to start recording a user’s movements are customisable. According to the University’s guidelines on the ethical conduct of research, we set these measures to capture the necessary details without recording the exact location of users. For this study, we set the application’s location accuracy to 10 m, and its threshold to start recording a trip to 10 m of direct distance movement. Therefore, the recorded data are always associated with 10 m of inaccuracy, while the first 10 m of each trip is also ignored. When a trip ends, the application automatically removes the last 10 m of the trip before storing it on the user’s smartphone.

Third, the application is designed to work offline after its user logs in for the first time. Therefore, all user movements are stored locally on the user’s smartphone. The user has complete control over uploading their trips on the server. The user can also select which days of their recorded activities to be uploaded on the server. As highlighted in the research participation information sheet shown to users when they register in the survey, a user can ask to remove all their data from the server at any time. The server is managed by the University of Queensland with very high security standards. Furthermore, the location data are always encrypted before being stored on the server.

The application is publicly available for personal evaluation. Furthermore, it can be used by other institutions and individual researchers. However, such a use is restricted to research purposes and should be reviewed by the University of Queensland’s Human Research Ethics Committee. The data are always uploaded by the application users to the server at the University of Queensland. A completely cleaned, de-identified dataset is delivered to external researchers ensuring the privacy of all participants.

We collected data on young people’s transport-related walking through this smartphone application in Brisbane, Australia during August–September 2014. The participants were mainly undergraduate engineering students at the University of Queensland. Direct recruitment of participants was through class contacts with students. These students could also nominate a relative or friend, external to the university, to participate in the data collection, and thus the sample includes non-students as well. Only individuals with a smartphone could participate in the study. No monetary incentive was provided for participation. In total, 199 participants were initially recruited for the study. These participants are not necessarily a representative sample of university students or the broader community. However, the recruited sample was sufficient to evaluate the applicability and usefulness of the proposed approach to objectively study young adults’ transport-related PA.

The participants were asked to use the application to collect personal mobility data for two weekdays. This required participants to keep the application running on their smartphones during all mobile activities. Overall, 170 participants completed the study, whom their travel data were processed to reveal the details of their transport-related walking, as explained in the next two subsections. Our follow-up investigation of the reasons for not completing the study by some participants revealed two major contributing factors. These factors include: a) concerns about the application’s battery consumption, and b) a lack of personally useful features in the application to motivate its usage.

### Data processing and analysis

The recorded raw data were cleaned, segmented into single modal trip-legs and verified in terms of the accuracy of the mode of movement for each trip-leg, as briefly explained in the next subsection. Only the data for the participants who reported their home addresses were used in the analysis of this study. This is because our objective is to investigate the timing and location of transport-related walking between a set of common activity nodes including each participant’s home location. The relevant details of the identified walking trip-legs were then calculated using the data recorded by the application. These details include the daily count as well as the actual length and timing of these trip-legs. The purpose of each walking trip-leg was also assigned to it, as identified by the respective participant for the corresponding trip.

The raw data collected by the smartphone application were cleaned and pre-processed in *RStudio* [44] using *R* language [45], adopting the algorithms proposed by Assemi et al. [46] and Safi et al. [43]. This is especially important as the smartphone application collects data on all movements of its users (including all modes of transport), while the focus of this study is on transport-related walking. Relying on the movement attributes estimated using the collected data (e.g., average speed, acceleration and idle times), the algorithms could split or merge trips’ data into single-modal trip-legs. Then, the walking trip-legs were extracted for the analysis of this study.

To investigate the patterns of transport-related walking trip-legs, we mapped these trip-legs to activity nodes, a high-level abstraction of location and activity. An activity node is a place that a given participant walks to/from and spends some time (at least 30 min) before going somewhere else. The activity nodes in this study are labelled based on the participants’ responses to the main type of activity they have performed in a given location. The activity nodes considered in this study for each participant include: home, education, work, shopping, eat/drink, health/wellbeing and changing transport mode. We identified the origin of each walking trip-leg by using the purpose of its preceding trip-leg (i.e., the previous day’s last trip-leg for the first trip-leg of the day). We used the corresponding trip’s purpose to identify the destination of each walking trip-leg. Therefore, while an activity node represents a specific location where a participant has walked to for a specific purpose (e.g., eating/drinking), it does not correspond to a certain point in the urban form and can point to a different location for each participant.

The resulting data were explored using descriptive statistics and different visualisation techniques in R to obtain insights about participants’ daily transport-related walking. To do so, we initially estimated the length and duration of each walking trip-leg based on the corresponding GPS records. We then examined the relative frequency of trips to and from particular nodes throughout the day; the proportion of circular trips (defined as trips that start and end at the same node); the time, frequency and average distance of trips between nodes most typical at each hour of the day. We also generated comprehensive di-graphs using the *igraph* package in R to investigate different aspects of participants’ transport-related walking trips throughout a day. These analyses are mainly to evaluate the usefulness and effectiveness of the proposed approach, while more advanced statistical analyses in future studies can reveal further insights from similar data.

## Results

### Descriptive statistics

To investigate the study participants’ characteristics and their transport-related walking, we initially present and discuss the descriptive statistics of the sample and the cleaned dataset. Table 1 summarises the socio-demographics of the participants. Overall, the data from 142 participants could be used after cleaning and pre-processing. As shown in Table 1, 108 (76.1%) participants were students and the rest were non-students; all of whom were employed (34 (23.9%)).

**Table 1:** Socio-demographics of participants (*n* = 142)

Characteristic	Categories	No of Participants	Corresponding Trip legs
Having access to a car	No	33 (23.2%)	97
	Yes	109 (76.8%)	325
Having access to a bicycle	No	129 (90.8%)	381
	Yes	13 (9.2%)	41
Age	20 years or younger	63 (44.4%)	172
	21–30 years	60 (42.3%)	198
	41–50 years	8 (5.6%)	24
	51 years or more	11 (7.7%)	28
Employment	Student	108 (76.1%)	333
	Working	34 (23.9%)	89
Weekly income bracket (AU$)	–	20 (14.1%)	54
	0–199	46 (32.4%)	134
	200–399	42 (29.6%)	128
	400–799	19 (13.4%)	63
	800-more	15 (10.6%)	43
Gender	Female	54 (38%)	151
	Male	88 (62%)	271
Household size	Up to 2	11 (7.7%)	28
	3 to 4	88 (62%)	260
	5 to 7	39 (27.5%)	119

Our sample is not representative of the general Australian population. For example, a large majority of the sample (123 participants, 86.6%) is younger than 30 years, while 19 participants (13.4%) are older than 40 years^2^. Of the participants, however, a majority has access to a car (76.8%); thus, our sample resembles the Australian population’s car ownership rates. While the participants’ characteristics limit the generalisability of our findings to a broader population, they are common among young adults who are the main target of this study.

As some participants had not provided their home addresses, which were required for the purpose of this study, only the data for the participants who had declared their home addresses (*n* = 142) were used in the analysis. The final processed dataset includes 422 person-day of observations. Overall, there are 630 transport-related walking trip-legs in the data set made by participants (*n* = 124). Eighteen participants in our final sample had not any significant transport-related walking. Table 2 presents the descriptive statistics of our sample’s transport-related trip-legs (including those without any significant walking trip-legs).

**Table 2:** Descriptive statistics of transport-related walking

Description	Min	Max	Mean	Std. Dev.
Total walking distance per travel day (m)	0	17722	1198	2148
Total walking time per travel day (min)	0	100.2	12.26	17.88
Number of walking trips per day	0	9	1.47	1.67
Total distance travelled per day (m)	86.45	278597	29466	35651

As shown in Table 2, on average, the participants travelled 29.47 km on a single day, of which 1.2 km is transport-related walking. The average duration of transport-related walking is 12.26 min for the sample. However, the transport-related walking behaviour varies greatly between the participants. This variation is demonstrated by the range (0 — 17.72 km) and standard deviation (2.15 km) of the total length of the daily walking trip-legs.

**Fig. 2:**
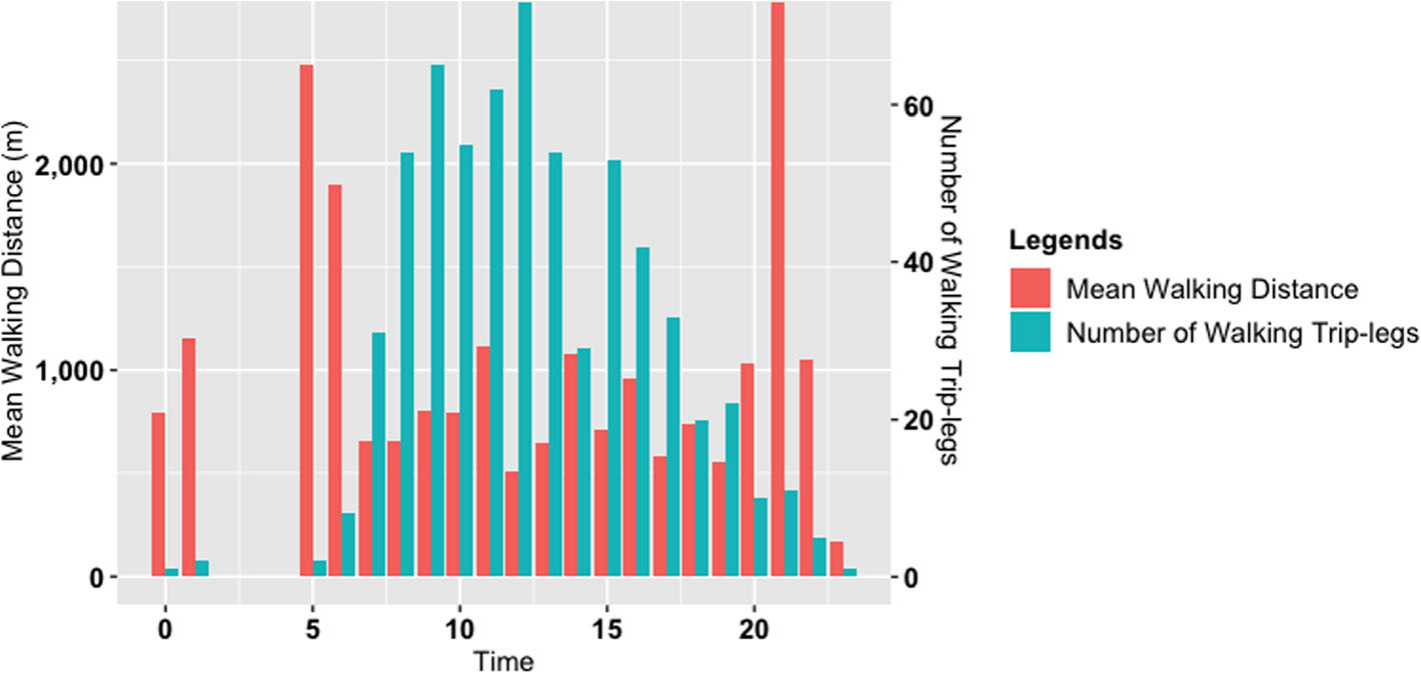
Mean walking distance and number of walking trip-legs in 24 h

Figure 2 shows the number and average distance of transport-related walking trip-legs at different times of a day for the whole sample. As shown in Fig. 2, the number of walking trip-legs is relatively low in the morning (before 7:00) and the evening (after 17:00). The number of walking trip-legs is relatively consistent between the two extremes, while it is slightly lower in the afternoon compared to the morning. This suggests that the participants tend to engage in a large proportion of incidental walking during normal working hours. Moreover, the lower number of walking trip-legs in the early mornings and late evenings can also be related to safety considerations and lighting. This finding suggests the need for holistic community-based policy responses that considers crime and disorder prevention and responses as well as safety interventions (e.g., effective lighting), in addition to the provision of footpaths and activity spaces. To fully understand such a potential relationship, however, it is necessary to conduct a rigorous investigation considering participants’ perceptions of safety and crime.

While fewer trips occur in the early morning (before 7:00) and late evening (after 20:00), the average length of walking trip-legs is usually longer at these times (as shown in Fig. 2). Between these two extremes, the mean walking distance is consistently around 800 m, although there is a drop at noon (around 12:00–13:00). Furthermore, the transport-related walking trip-legs are on average slightly shorter in the afternoon compared to the morning. This can be attributed to the temperature and weather conditions during afternoon, highlighting a potential need for shades to enhance walkability.

### Travel purpose and walking

**Fig. 3:**
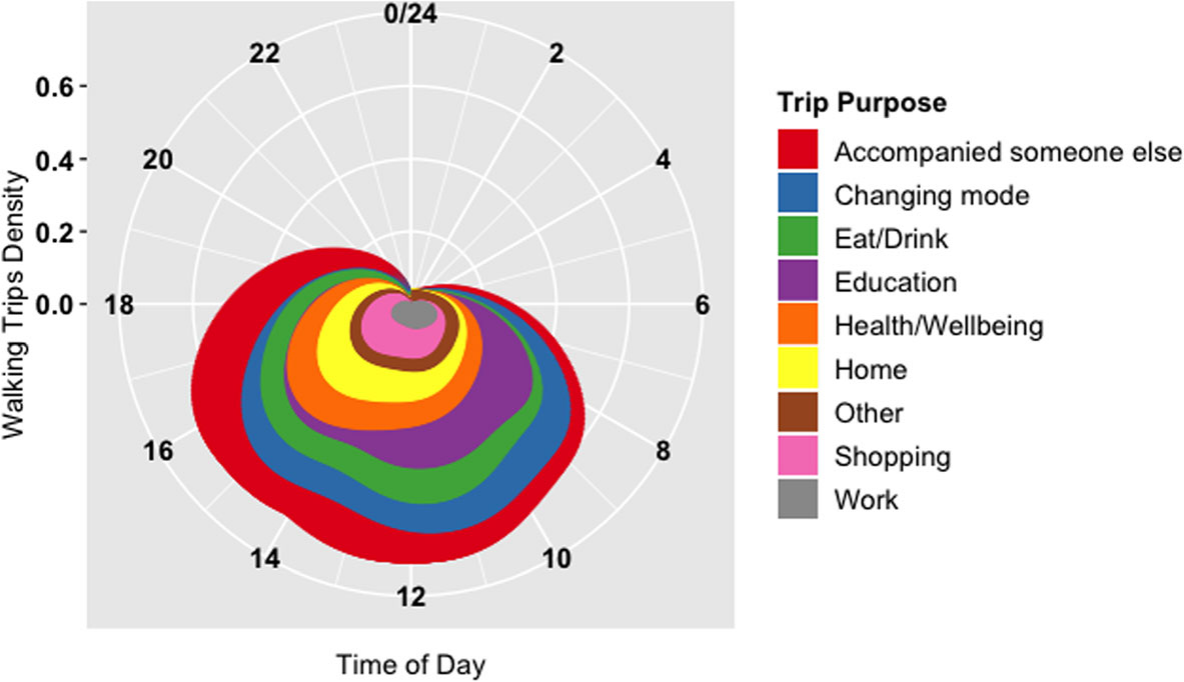
Distribution of walking distance based on time and trip purpose

An exploratory investigation of the travel purposes identified by the participants reveals interesting patterns in the participants’ transport-related walking. Figure 3 shows the distribution of the share of walking distance based on trip purpose. As shown, most transport-related walking occurred between 7:00 and 19:00 (i.e., working hours). While the density of walking trip-legs for “work” is relatively evenly dispersed throughout the day, the density of walking trip-legs for “education” is significantly larger in the morning and the density of walking trip-legs for “shopping”, “home”, “health/wellbeing” and “accompanying someone else” is much larger in the afternoon, compared to other times throughout the day. The density of walking trip-legs for “eat/drink” and “changing mode” is greatest between 9:00 and 16:00, with its peak occurring around noon (i.e., lunch time) for the former.

In summary, morning trips are often associated with obligatory activities (e.g., education and work), while afternoon trips are usually associated with third places and non-obligatory activities (e.g., shopping, health and wellbeing, and accompanying others — social). Moreover, there is a higher likelihood of walking longer distances in the morning as a part of a trip-chain, given the larger density of walking for changing mode between 8:00 and 12:00.

### Walking and activity nodes

**Fig. 4:**
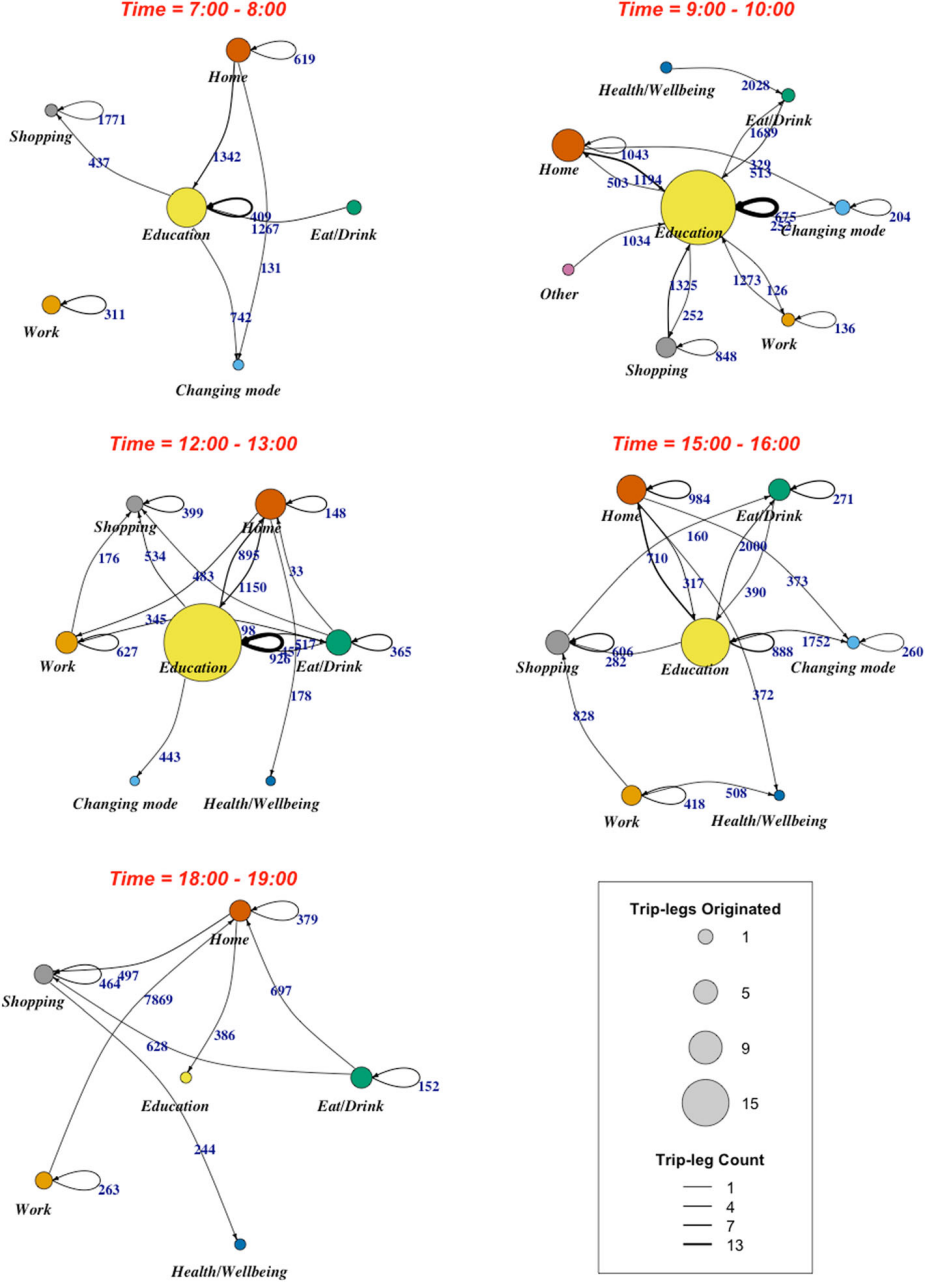
Walking trip-legs between activity nodes across a day

Figure 4 delineates walking trips between different activity nodes at different times of the day (i.e., 7:00–8:00, 9:00–10:00, 12:00–13:00, 15:00–16:00 and 18:00–19:00) for the whole sample. The nodes and paths in this figure illustrate activity nodes and walking trips between them, respectively. The size of each activity node indicates the total number of transport-related walking trips that originated from that activity node (i.e., the larger each node is, the more walking trips have originated from that node). The width of each path represents the number of walking trips, while the path labels show the mean walking distance between the respective origin and destination activity nodes.

Figure 4 demonstrates that between 7:00 and 8:00 in the morning, most transport-related walking trips originated from either home or education nodes. This is the time that most participants were either leaving their home or walking from one building (on-campus college) to another to attend their classes at the university. The three largest mean walking distances during this period correspond to home–education (1342 m), shopping–shopping (1771 m), and eat/drink–education trips (1267 m). This indicates a high likelihood of long walking trips between early morning eating/drinking or home and the university for our study participants.

In the 9:00–10:00 time period, most frequently, walking trips continue to originate from the home and education nodes. Shops are also a frequently occurring origin node of walking trips during this time period. Notably, the number of walking trips originating from the education node is relatively high when compared to home and shops between 9:00 and 10:00 in the morning. This can be attributed to walking trips between buildings on the university campus. A further indication of intra-campus travel is the high number of circular trips occurring at the education node during this time period. Indeed, these trips represent the most frequent type of walking trips during this time period. Home–education and shopping–education trips are also frequent between 9:00 and 10:00 in our sample. The largest mean walking distances were associated with trips between education–eat/drink (1689 m); shopping–education (1352 m) and health–eat/drink (2028 m), suggesting that compared to intra-campus trips, those involving non-educational and potentially off-campus activities also require more walking throughout morning hours.

Patterns in transport-related walking trips during the midday time period (12:00–13:00) are similar to those displayed in the morning hours with a few notable differences. First, the workplace, along with home and education, generates the greatest number of walking trips during this period. As in the morning hours, education–education and home–education trips constitute the majority of walking trips, however, return trips between the education node and home (education–home trips) also start to become more frequent during the midday period. Walking trips between home and the educational node and those between the educational node and eating/drinking venues (off-campus) tend to be longer than those contained within the educational node during this time period.

The largest shift in patterns of transport-related walking trips was evident in the afternoon between 15:00 and 16:00. Most frequently, during this time period, transport-related walking trips are between education and home signifying the end of the study day. 15:00 is the end of school time in Australia — symbolically recognised as the end of students’ day. This may influence routine activities of the university students as well, if they are (a) first years and perhaps still engrained with the 15:00 finish times; and/or (b) have a part-time job that requires starting for the after school busy period. Circular trips are also common during this time period. The largest number of circular trips are generated at home, education, shopping and eat/drink nodes. The longest walking trips during this period were between home and a mode change node (2000 m); an education node and a mode change node (1752 m) as well as home-based circular trips (984 m). Mode change nodes indicate points of transport mode transfer, for example, walking to train travel or walking to driving. This node becomes an interesting contributor to walking trips during this period, highlighting the importance of better understanding trip chaining as a potential opportunity for increasing transport-related walking. Chaining refers to the use of multiple modes of transportation to achieve a singular journey. For example, walking from home to a train station and then catching a train to work.

Patterns of transport-related walking during the evening period, between 18:00 and 19:00, are distinct from daytime patterns in a number of ways. As expected, home, eat/drink and shopping nodes generate the majority of walking trips during this period. Most frequently, walking trips are between home and shopping nodes or comprise circular trips based around shopping or eating and drinking activity nodes. Walking trips between work and home are less frequent during the evening, but when they do occur, they are longer (7869 m on average). Walking trips from eating and drinking nodes to shops or home (628 m and 697 m, respectively) also have high mean distances compared to other trips during this time period. Overall, walking trips are much shorter during this period, compared to all other times of the day. This may be related to darkness and feelings of safety at this time of the day.

**Fig. 5:**
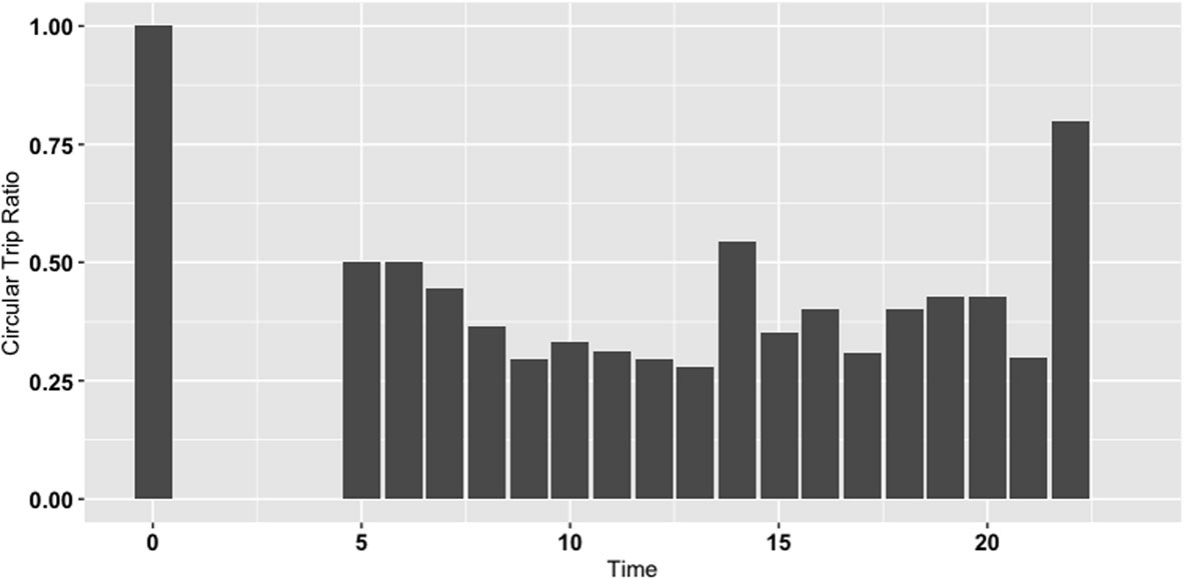
Ratio of circular trips (from one activity node to itself)

Finally, Fig. 5 shows the ratio of circular walking trips to all walking trips at different times of the day for the whole sample. As shown, there are more circular trips early in the morning (between 5:00 and 7:00) as well as late in the evening (18:00 onward). This indicates that the participants were more likely to walk from their current location to the same location (potentially their home) or a different location with the same functionality (e.g., two different shops for shopping) during these times. An exception to this trend is a high share of circular trips around 14:00. This is potentially when people walk to purchase a coffee or lunch without spending much time for eating/drinking in the same place, and they go back to their origin node.

## Discussion

### Key findings

In this study we proposed and investigated potential advantages of a unique data collection technique to study people’s transport-related, incidental walking. Accordingly we developed a smartphone application, through which we collected accurate, fine-grained data on mobility patterns of a sample of mostly university students, focusing on their transport-related walking during a regular daily routine. As previously discussed, an understanding of such patterns can inform urban design and indicate where PA-facilitating urban form features such as sidewalks and shades can have the greatest potential to impact population health.

We identified eight main activity nodes which operate as transport-related walking generators. We investigated the number and average distance of walking trip-legs for our sample between these nodes at different times of the day to reveal where and when transport-related walking is most likely to occur.

The first key finding of this study relates to primary walking trip generating locations. While to date, most research and interventions concerning PA (especially in young adults) have focused on the residential neighbourhood [25, 47, 48], the results presented here identify the educational node — which in other samples may be represented by the workplace — as an equally important node for generating walking trips. This is likely related to the large proportion of hours routinely spent at one’s place of education or work. Furthermore, the hours when individuals are at education or work tend to be during the daylight — in contrast to the time spent at home, which also encourage walking trips.

This pattern of routine activity is also related to our second key finding that transport-related walking trips are greater during the daytime, whereas at night time walking trips tend to be less likely as expected. Although this finding is not surprising, it highlights the potential role of feelings of safety in being more active, in line with the findings of previous research (e.g., [49]).

Our third and final key finding is the prevalence of ‘chained’ trips in the afternoon period. Research, planning and management of transport have often focused on single modes of transport (e.g., roads, ferries, buses, or rail) [50, 51]. This segregation across modes is also evident in resource allocation and service delivery. Yet, we demonstrate here that multi-modal trips are common in the afternoon period. Given that these trips tend to occur in the afternoon, when people are likely to have completed their obligatory tasks and are heading home, they may provide great opportunity for increasing transport-related PA. Unlike during the morning hours when people are time-constrained, they often have greater flexibility on the home-bound journey to increase their PA by getting off the bus a station earlier and walking the remainder of the distance home or walking to the shop rather than walking to the car and driving.

### Implications

This paper has implications for both researchers and practitioners, as the proposed approach of data collection and the insights obtained from the data collected through this approach can improve our understanding of young adults’ opportunities for PA. These opportunities, along with the studied barriers and facilitators play an important role in developing public health policies, planning urban form and designing transport networks [52].

The findings of this study highlight how people’s daily PA is largely governed by biological necessities (eating and sleeping) and routine activities or obligatory behaviours stemming from social-behavioural norms and expectations. Based on these findings we suggest increasing daily PA through encouraging greater transport-related walking. This is especially important, as changes in mobility are associated, at least in part, with changes in behavioural norms, especially those around working hours, commuting distances and technology [53–55]. Therefore, increasing transport-related PA can play a role in addressing the negative side effects of rapid urbanisation on people’s health and wellbeing.

We acknowledge the complex and persistent nature of reduced PA among certain groups and populations, and we do not propose that transport-related PA alone is the answer to the problem. Rather, in this study we explored patterns of transport-related walking throughout the day, and drawing on the results, we suggest that increasing opportunities for individuals to engage in this form of exercise is one of a number of approaches, that if applied simultaneously, could start to address the reduced PA problem. The benefits of increased daily walking for individual health and wellbeing are well documented. Built on the findings of previous research (e.g., [42, 56–58]), we argue that PA incorporated into routine daily activities, in particular transport-related PA incorporated into the daily commute to obligatory activities, is an effective approach to increase population levels of physical activity. Encouraging more frequent transport-related walking activity is a relatively cost-efficient intervention that can be developed incrementally over time.

Finally, the findings of this study highlight the need for developing localised multipurpose nodes that encourage walking by making it possible for residents to engage in ‘functional’ walking trips to access shopping, eating/drinking and transport nodes.

### Limitations and future research outlook

This study has some limitations that can be addressed in future research.

First, this study has relied on a small sample to collect data required for evaluating the feasibility and usefulness of the proposed approach. As mentioned earlier, this study has focused on evaluating a smartphone-assisted method for transport-related PA data collection and analysis, with low cost and high accuracy. This study does not intend to draw generalisable conclusions from the data. To further examine the usability of the proposed approach and to achieve generalisable findings, larger samples should be recruited in future studies, which are representative of populations of interest.

Second, this study investigated transport-related walking between activity nodes, an abstract representation of activity–location. Therefore, the urban form and its physical attributes have not been considered as such in this study. To better understand the impact of urban form and the corresponding design factors which can influence people’s transport-related PA, it is necessary to evaluate potential associations between location-specific urban design attributes and transport-related PA in future studies.

Third, the findings of this study suggest potential impact of weather conditions on the participants’ transport-related walking — only throughout a normal day. However, weather conditions were not specifically considered in this study; Therefore, it is insightful to collect data in different weather conditions and explicitly examine any relationships between such conditions and people’s transport-related PA in a future investigation.

## Conclusion

This study demonstrates the utility of a unique approach toward transport-related PA data collection and analysis. More specifically, this study proposes and evaluates the advantages of using a smartphone application for collecting accurate, fine-grained, and objective data on people’s transport-related walking. Furthermore, it explores transport-related walking patterns through the use of di-graphs, which in particular, contributes to a better understanding of transport-related PA and opportunities for intervention to increase incidental walking. The case study presented in this paper acts as a proof-of-concept showing the feasibility and usefulness of the proposed approach.

The findings noted in the previous section illustrate how collecting this sort of fine-grained information on a range of samples can inform urban form that enhances walkability at locations that are likely to generate walking trips, as well as between activity nodes that are most commonly connected by walking trips. Further, this study’s insights into patterns of transport-related walking activity can help to shape public education and awareness campaigns that aim to encourage walking trips throughout the day by suggesting locations and times of the day when engaging in these forms of exercise is easiest and least intrusive. Finally, this study’s finding about the potential role of multi-modal trips in encouraging transport-related walking provides an outlook for future research about preceding and following trips of walking trips, in terms of mode and purpose, to better understand potential opportunities for transport-related PA that can be generated through these trips.

## Data Availability

The datasets generated and/or analysed during the current study are not publicly available to preserve participants’ privacy, but are available from the corresponding author on reasonable request.

## Abbreviations

AU$: Australian Dollar
GPS: Global Positioning System
GSM: Global System for Mobile communications
PA: Physical Activity

## Footnotes

1 The app is available on App Store (https://apps.apple.com/au/app/atlas-ii/id826111885) for iOS devices and Google Play (https://play.google.com/store/apps/details?id=au.edu.uq.civil.atlasii&hl=en_US) for Android devices.

2 Our sample did not include any participants between 31 and 40 years old. This can be attributed to our recruitment procedure. As mentioned in the previous section, the participants were recruited from undergraduate engineering students, while they could also nominate their relatives to participate in the study. Given the high likelihood of nominating their parents in the latter case, it is very likely that such a relative is older than 40 years.
